# Gastronomic Curiosity and Consumer Behavior: The Impact of Television Culinary Programs on Choices of Food Services

**DOI:** 10.3390/foods13010115

**Published:** 2023-12-28

**Authors:** Bożena Gajdzik, Magdalena Jaciow, Radosław Wolniak, Robert Wolny

**Affiliations:** 1 Department of Industrial Informatics, Silesian University of Technology, 40-019 Katowice, Poland; 2 Department of Digital Economy Research, Faculty of Economics, University of Economics in Katowice, 40-287 Katowice, Poland; magdalena.jaciow@ue.katowice.pl (M.J.); robert.wolny@ue.katowice.pl (R.W.); 3 Faculty of Organization and Management, Silesian University of Technology, 44-100 Gliwice, Poland

**Keywords:** gastronomic consumer, consumer behaviors, television cooking, food choices, culinary programs

## Abstract

In highly developed countries, more and more people use culinary services. Cooking at home, for the family, is giving way to culinary services. Consumers either order food home or use the offers of restaurants and bars. Consumers’ choice of culinary form may be influenced by cooking television programs. Many TV stations broadcast cooking programs. This study examined the impact of television culinary programs on consumer behavior in the restaurant services market. The article examines the interplay of emotional responses, personality traits, and culinary preferences to understand how TV cooking programs influence dining decisions. The study was conducted using the CAWI method, which involved 742 respondents. The study, conducted between May 2021 and April 2022, was addressed to people who visited restaurants presented in the Polish culinary TV show titled “Kitchen Revolutions”. The study revealed that almost 3/4 of the respondents chose restaurants based on the program’s recommendations. Although there was a strong emotional connection with this program—nearly half of the respondents expressed delight in the taste of snacks and main courses—this did not always translate into an increased frequency of eating meals away from home. Only every third respondent said that the program influenced their gastronomic behavior. The research hypotheses examined the extent to which culinary TV shows influence the frequency of visits to restaurants, the perceived quality of life, and the influence on consumers with specific personality traits. The results partially supported the hypothesis that cooking programs on television encourage people to eat out more often, but the perceived impact on quality of life and on some personality traits was less clear. The article contributes to the understanding of consumer behavior in the food service market by highlighting the complex dynamics of emotional reactions, personality traits, and the impact of culinary television programs. The findings have practical implications for the restaurant industry, suggesting a focus on emotional impact, food quality and presentation, and targeting marketing strategies towards consumers who are open to new experiences and ready to experiment.

## 1. Introduction

In recent years, many television stations have been offering their viewers programs that teach cooking step-by-step or show changes made in restaurants by prestigious chefs [1]. The interest of viewers in such programs is increasing, as these productions provide not only culinary recipes but also an element of competition among participants, as well as insights into restaurants with excellent cuisine [2]. Television stations often invite prestigious chefs to host these programs. The constantly evolving formula of these shows, along with old and tested concepts, often based on foreign licenses, attracts many viewers to their screens [3]. A notable phenomenon of culinary programs broadcast on television is that people are learning the art of cooking [4]. There are also consumers who utilize the services of restaurants featured in these television programs to try new dishes [5]. People are curious about new flavors or are so strongly inspired by the changes in restaurants shown on TV that they want to personally check the results of these changes. For many people, visiting restaurants that have undergone transformations witnessed by TV audiences becomes a passion and a form of gastronomic tourism [6,7].

There are many countrywise differences in the case of cooking programs. In Italy, cooking programs might heavily focus on preserving and celebrating traditional culinary heritage, with an emphasis on regional ingredients and authentic recipes. Viewers may be more interested in learning classic Italian cooking techniques [8]. In the United States, the celebrity chef culture could be particularly pronounced, and viewers may be drawn to cooking shows featuring well-known chefs. The competitive element and personal stories of chefs may be a significant factor in the popularity of such programs [9]. In France, cooking shows may highlight the importance of locally sourced, high-quality ingredients. Viewers might be more inclined to follow programs that emphasize the use of fresh and seasonal produce in their cooking [10]. In Spain, there may be a trend of viewers engaging with cooking shows that explore culinary tourism, featuring different regional cuisines and the unique flavors associated with various Spanish provinces [11]. In the United Kingdom, cooking programs may reflect the multicultural nature of society, incorporating a wide range of global cuisines. Viewers might be interested in exploring diverse flavors and cooking styles from around the world [12].

Restaurateurs are increasingly having to vie for consumers of their dishes, employing more sophisticated methods of acquisition [3,13]. The competition for customers is becoming more refined, and the analysis of consumer behavior more detailed, as many restaurants see this aspect of marketing activities as a potential for success in a competitive market [14]. The prestige of a restaurant guarantees the elevation of the consumers’ status and focuses all efforts on them to ensure maximum satisfaction [15]. The needs, preferences, and desires of consumers are not the only determinants of a restaurant’s offerings, as new forms of reaching customers are becoming increasingly important [16,17]. One such method is registering the restaurant for a television program to become a filming location or a subject for transformation [18].

This publication attempts to elucidate the impact of a culinary television program on consumer behavior in the gastronomic services market. This research is based on the popularity of the “Kitchen Revolutions” program. This program has been broadcast on Polish television, on the commercial station “TVN Warner Bros. Discovery”, since March 2010 and is hosted by the restaurateur Magda Gessler. The program’s popularity in Poland led the authors of this publication to deem it appropriate to introduce into their research an index named after Gessler, referred to as “Gesslerism”. This index is developed by the authors of the publication and, in a detailed way, described in the following part of this paper. Restaurants with culinary and organizational problems, leading to a decline in customer numbers, apply to the program. After each “revolution” or corrective actions taken by the program’s host, the number of customers increases.

Much like Gordon Ramsay’s programs that follow a similar format, “Kitchen Revolutions” involves restaurants facing culinary and organizational challenges that have resulted in a decline in customer numbers [19]. These establishments seek the assistance of the program, which leads to a series of culinary interventions and corrective actions facilitated by Magda Gessler. Subsequently, the program documents a notable increase in the number of customers following each “revolution”, showcasing the positive impact of the interventions on consumer engagement [20].

The paper consists of six main parts: Section 1: Literature Review and Conceptual Research Model. This section provides a concise description of the research context and presents the conceptual research model. Section 2: Materials and Methods. It briefly describes the main methods and hypothesis development. Section 3: Results. This section summarizes the main findings of the realized field research. Section 4: Discussion about the Results of the Research. Section 5: Conclusions. This includes the main interpretations of the realized research and discussion. Section 6: Limitations and Future Research.

## 2. Literature Review and Conceptual Research Model

### 2.1. Key Concepts

In a highly developed society, also known as a consumer society, people’s behaviors are largely dependent on the influence of a trend called consumerism. Consumerism is a characteristic of every developed society [21]. In recent decades, mass media (radio, television, the Internet, etc.) have had a significant impact on the development of consumption [22]. The media participate in creating the world, including consumer habits [23]. The culture of consumption places humans in a world of culinary values. These values form the basis of human action in the consumption of goods and services. The concept of consumption values, from the 1970s [22] to today, has changed significantly. Nowadays, the value of consumption is multifaceted and can take the form of an object of interest from individual units or social norms meant to guarantee the interest of specific groups or the entire society [23]. According Tanrikulu [24] the concept of values is important for marketing research. Generally, consumers prefer to buy products with the highest perceived value [25,26]. Consumption values can produce an effect through customers’ attitude. Consumption attitudes influence purchase decisions, such as the type of clothing, food, choice of car, and how leisure time is spent. Values are indicators of consumer and social aspirations. Aspirations arise at the interface of values with the world view considered in terms of possibilities, chances, and hopes [27]. The reception of this image is greatly influenced by media messages. Consumers increasingly build their attitudes detached from the real world, focusing more on the virtual world. Such an attitude is a result of the development of the Internet and computer technologies. Therefore, it is not surprising that media recipients want to check the world view presented on television, the Internet, and other media. Curiosity about new places, experiences, and people, etc., enhances aspirations and vice versa; consumer aspirations amplify their curiosity to explore the world. Curiosity is a determinant of changes in consumer behavior values. Under the term “curiosity”, we understand the emotional impulse to consume, to learn, to act, etc. [28]. Satisfying curiosity has symbolic significance. The consumer assigns symbolic meaning to a good, which can be pleasure (in line with the idea of hedonism), “connoisseurship” in line with the idea of sublimation, and other features belonging to the category of individualism [23,29].

Cooking shows generate consumer curiosity, creating a tension linked to the desire to discover new culinary experiences or restaurants featured in these television programs (Table 1) [27,28]. Consumers eager to visit restaurants transformed by television shows demonstrate their position in the gastronomic market in this way [29,30]. Their curiosity leads to the consumption in restaurants altered by television programs. In doing so, consumers aim to diversify their existing individual styles, showcasing their interest in novelty, taste of dishes, originality of dishes, restaurant decor, etc. [31]. Cooking is presented in various forms of television programs, such as travel shows, lifestyle shows, reality programming, game shows, renovation programs, gastronomic visits, programs about starred restaurants, etc. [31,32,33].

The United States has the most cooking television programs. About half of Americans watch cooking shows ranging from occasionally to very often [34]. Over half (57%) of the viewers say they have purchased food as a direct result of something they have seen on a cooking show, 36% say they have purchased small kitchen gadgets, and 24% say they have purchased cookbooks [35]. The hosts of cooking television programs often attract the attention of viewers, and they are not always master chefs but sometimes actors, journalists, and other personalities regarded as celebrities [1,2,3]. Celebrity chefs play a vital role in obtaining viewership for food-related television programs and can exert significant influence over their viewers [9,15]. Chefs act as culinary teachers or serve as entertainers based on their television persona and lifestyle portrayal that audiences can relate to [32].

Television cooking shows are appealing to various age groups in society. TV is the primary communication vehicle, especially among lower-income populations, capable of transmitting information from education to entertainment [13]. Watching TV is the dominant recreational pastime across all age groups, particularly among children and adolescents [14]. According to the Harris Poll [35], cooking shows are attractive to both young and old people.

Current literature reviews show that food programs may impact viewers similarly to television food advertisements [36,37]. Cooking programs can influence the foods purchased and the types of dishes cooked by viewers. Some cooking television programs promote health-conscious messages, while others do not [35,36,38]. Both types provide inspiration to their viewers on cooking and have the potential to influence the purchases of those watching [34,35]. Cooking shows may influence the dietary habits and choices of viewers, given their prevalence on television and the popularity of celebrity chefs.

There are also many studies on the impact of television programs on viewer behavior. Table 1 presents a compilation of selected significant studies concerning the influence of television programs on consumer behavior.

**Table 1:** Selected studies on the impact of TV programs on culinary choices.

Source	The Aim of the Study	Research Sample	Effect
Dixon et al., 2007 [39]	The effects of television advertisements for junk food on children’s food attitudes and preferences	19 grade 5 and 6 students from schools in Melbourne, Australia	Positive if we advertise nutrition food
Ejsymont et al., 2010 [40]	Assessment of the impact of television culinary programs on shaping the nutritional behavior of viewers	134 Internet users	Positive
Boulos et al., 2012 [41]	How television is influencing the obesity epidemic	Obese children in the USA	Negative
Lane and Fisher, 2015 [1]	To investigate the exposure of a student population to celebrity chef television programs and to assess the influence these figures have and how they are perceived	A survey through an online questionnaire distributed at Bath Spa University—238 persons	Positive
Villani et al., 2015 [2]	To investigate people’s attitudes and beliefs about popular television cooking programs and celebrity chefs	207 participants undertook the questionnaire	Neutral
Temeloğlu and Taşpınar, 2018 [6]	To determine the relationship between the cooking shows on TV and the people’s intention of participating in gastronomic tourism	391 tourists that participated in gastronomic tourism	Positive
Ngqangashe et al., 2018 [42]	The effect of TV cooking show consumption on children’s food choice behavior	A sample of 85 children (32 girls and 48 boys) from two schools with comparable student profiles in terms of sociodemographic characteristics	Positive
Kizileli et al., 2019 [33]	To examine how celebrity chefs influenced the nutrition planning of their viewers	The study population was adult television viewers (≥18 years of age)—244 female and 144 male	Neutral
Folkvord et al., 2020 [43]	To test the effects of a cooking program on healthy food decisions	Class settings in 5 different schools	Positive if we advertise nutrition food
Fraga et al., 2020 [44]	To verify the effects of buying television-advertised food on schoolchildren eating behavior	797 children were evaluated, the mean age was 9.81 (0.59) years, 50.7% were female, and 32.4% were overweight	Neutral

Source: own study based on [1,2,6,33,39,40,41,42,43,44].

A research conducted in Poland [40] by Ejsymont et al. focused on assessing the impact of television cooking programs on shaping the eating behaviors of viewers. The study was conducted using a survey method among 134 Internet users. The questions concerned the types of cooking programs watched, the factors influencing their choices, evaluation of dishes presented in the programs, and changes in eating habits under the influence of television cooking programs. The researchers found that television cooking shows influenced the eating behaviors of viewers, including in terms of food choices and introducing innovations into the daily dietary menu. Ejsymont et al. concluded that cooking programs influence the shaping of eating behaviors and, thus, can be a good tool for implementing nutritional education [40].

The scope of research on consumer awareness in the gastronomic services market is constantly expanded to include more areas of influence, such as “ecological cooking”, “dietetic cooking”, and “healthy cooking”. Television requires no literacy or user skills, caters to an audience of all ages, is present in almost every household, and maintains its influence despite the growing prevalence of Internet use. Consequently, television broadcasts have tremendous power in shaping the food and nutrition knowledge of consumers [45,46,47,48]. Correspondingly, there is a rapid increase, in both quantity and quality, in cooking shows and other food- and nutrition-related shows broadcast on television worldwide [32,49].

In the realm of gastronomic consumption, individuals strive to create a unique, personal consumption style, marked by individual character. The motive behind such behavior is twofold: on the one hand, there is a pursuit of personal identification (“I want to distinguish myself from others”), and on the other hand, a pursuit of group identification (“I also belong to this group of people who have visited restaurants after changes”). Such behavior can be part of gastronomic tourism [50]. Gastronomy experiences are becoming a fundamental factor influencing the decision to choose a travel destination, as well as a crucial factor in shaping tourists’ satisfaction with their overall travel experience. Good restaurants add value to a place visited by a tourist. Gastronomy tourism is a way for a destination to prosper by showcasing its local cuisine cultures and local products, and contributes to the destination’s brand image [51]. Broadly speaking, food trips may include visits to local farmers and producers, local food fairs and markets, gastronomy events and festivals, participation in cooking masterclasses, and tasting of meals and beverages [7].

The television popularity of local restaurants attracts customers [52,53]. For many consumers, being in restaurants known from television is a very important element of culinary experiences, most often acquired during holiday trips [54,55]. Hall et al. [56] indicated that “gastronomy experiences are a window to the culture of the destination”. Consequently, studies suggest that culinary experiences contribute to shaping one’s personal satisfaction with the trip [50].

Julia Child was a pioneer of television cooking programs worldwide, beginning their production in the United States in the 1960s [57]. The popularity of television cooking shows is also confirmed by viewership rankings in Poland. The peak of popularity for television cooking programs in Poland was at the beginning of the 21st century. In 2016, the programs “MasterChef Junior” and “MasterChef” reached an average minute rating (AMR) of over 5 million viewers, surpassing the viewership of the UEFA Euro 2016 football championships broadcast on TVP1. These programs are among the most watched on the “TVN Warner Bros. Discovery” network [54,58].

Television cooking shows significantly influence many people’s choice of gastronomic venues. There are numerous ways these programs can affect consumers’ decisions about dining out [39]. Cooking shows often promote specific restaurants, cafes, or culinary venues. Showcasing tasty dishes and unique culinary experiences can attract viewers to these places. Television cooking programs can inspire viewers to search for new and unusual flavors. After watching a dish prepared by a famous chef, viewers may be motivated to try a similar dish in a local eatery [59]. These programs often educate viewers about various world cuisines, cooking techniques, and ingredients. This can make viewers more discerning and interested in culinary diversity, encouraging them to experiment with different venues. Cooking shows influence culinary and nutritional trends, such as the popularity of vegetarian or vegan dishes or cuisine from specific regions [60]. Restaurants offering these dishes may attract more customers. Some cooking shows include segments where experts critique dishes and culinary venues. These reviews can influence customers’ decisions on where to dine. Cooking shows often feature chefs and presenters traveling to different regions and sampling the local cuisine [4,47]. This can inspire viewers to seek similar experiences in local restaurants [5,13]. Some cooking shows collaborate with brands and food products, promoting them within the show [61]. Viewers may be inclined to visit gastronomic venues that use these products [62]. Viewers often admire and value chefs who appear in cooking programs. Restaurants run by such renowned chefs can become popular and attract many customers [63,64]. Table 2 compiles factors influencing choice.

Especially celebrity chefs can have a significant influence on people’s choice of gastronomic local [65,66,67]. Celebrity chefs frequently appear on television cooking shows, culinary competitions, and interviews [15]. Their media presence ensures that their recommendations and restaurant choices reach a broad audience, increasing the visibility of chosen gastronomic locals. Additionally, some celebrity chefs have their own restaurant chains or culinary brands. When these chefs endorse or operate a restaurant, it often attracts fans and food enthusiasts who want to experience the chef’s culinary vision firsthand [16].

They are known for pushing culinary boundaries and setting food trends. People are often curious to try the latest food trends or creations made famous by these chefs, leading them to the associated restaurants [17]. They also often have a strong presence on social media platforms, where they share insights, recipes, and restaurant recommendations. This digital presence further reinforces their influence over their followers’ dining choices [17].

Viewers may feel a personal connection with celebrity chefs through their storytelling, culinary journeys, or relatable personalities. This connection can motivate individuals to support the chef’s recommended restaurants. Celebrity chefs frequently host special events, pop-ups, or collaborations with restaurants [68]. These exclusive dining experiences can create a sense of excitement and urgency among fans to secure a reservation. They are typically associated with high-quality culinary experiences. Diners often expect exceptional food, service, and overall dining experiences when visiting restaurants endorsed by these chefs [69].

In Poland, the most popular culinary television program is “Kitchen Revolutions”. This show has been broadcast since March 2010 on the “TVN Warner Bros. Discovery” network. It is hosted by the restaurateur Magda Gessler and was modeled after “Kitchen Nightmares” aired in the United Kingdom, hosted by Gordon Ramsay. The aim of the program is to implement changes necessary to increase the turnover of restaurants applying to the program (including changes in the menu, interior design, and staff management). The mission of the program is not only to attempt to save restaurants but also to convey knowledge about preparing dishes, mainly regional cuisine [54,70]. The “Kitchen Revolutions” program has been invariably popular among viewers for 13 years. Since 2010, 27 seasons of the show have been produced, for a total of 368 episodes. The eccentric host Magda Gessler has already helped hundreds of restaurateurs whose restaurants were on the verge of bankruptcy. Despite the passage of time, the TV show still has high viewership. On Thursday evenings, there are an average of more than 1 million viewers over the age of 4 and more than half a million viewers in the 20–54 age group [70].

In contemporary scholarly discourse, various factors influencing the demand for gastronomic services have been identified, leading to attempts at a conceptual modeling of this domain. Notable examples include “The Five Aspects Meal Model” [71] and a model integrating gastronomic tourism [72]. Notwithstanding these developments, there remains a gap in the literature concerning models that incorporate the influence of culinary television programs. 

The scholarly consensus indicates that the demand for gastronomic services is articulated through consumers’ expressed intentions to utilize these services, stemming from identified needs and underpinned by tangible purchasing power. This demand is multifaceted, subject to a plethora of influences that can be categorized in diverse manners [73]. A commonly employed categorization in the literature distinguishes between economic and noneconomic factors [74]. Economic factors encompass consumer income, the availability of gastronomic services, the supply of food products (outside the service context) deemed substitutable for such services, and the pricing of both gastronomic services and these substitutable food products [75]. In contrast, noneconomic factors include demographic variables (population size and composition by gender and age), social aspects (education, employment status, nature of work), sociocultural dimensions (customs, traditions, habits), sociopsychological elements (needs/motivations, preferences, fashion trends, mimicry, social pressure, expectations, propensity for experimentation, personality traits), and technical and technological factors (usage of mobile devices, Internet access) [76]. 

A key factor in the choice on the gastronomic services market is their quality [77,78]. For many restaurant owners, the quality of gastronomic services is solely associated with menu items and service. However, most of them are not aware of the complexity of this issue. The perceived quality of services in this industry is composed of many elements: the atmosphere of the place, the development and equipment of the premises, and all sensations and feelings associated with the guests’ stay (comfort, privacy, music, colors, scents, staff’s mood) [79]. It is important to realize that every customer is different, and they have varying needs, desires, and expectations. Just as various motives drive a customer to choose one establishment over another, so too will the reasons why they return to that venue or, worse, choose not to return. It is important to remember that the quality of the served dishes affects the health of consumers [80]. In research [81], it has been determined that the following factors influence the purchasing decisions of gastronomic service customers: the quality of the served meals, features of the gastronomic venue, service quality, and price. The concept of meal quality was mainly associated with its sensory attributes and, to a lesser extent, with the quality of used food raw materials and the variety of offered dishes. Quality is also significant in the customer service management model in gastronomic establishments [82]. The author of the model discusses ways to improve the various elements of service quality to reflect the expectations of service recipients and presents tools to assess the level of guest service, in terms of technical (organization, information about the gastronomic service), functional (staff’s attitude towards the restaurant customer), quality culture/quality without obtrusiveness, elegantly addressing complaints, etc. 

Additionally, culinary television programs have emerged as a novel determinant in consumer behavior within the gastronomic service market [83]. 

### 2.2. Conceptual Model

**Figure 1:**
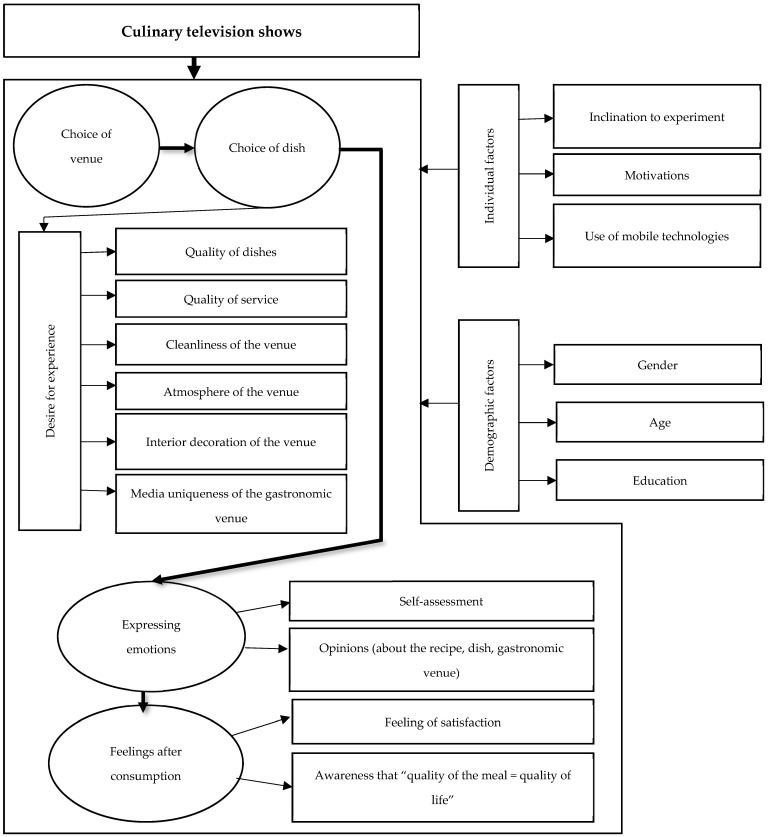
A conceptual model of the influence of culinary television programs on consumer behavior. Source: own study based on [74,75,76,77,78,79,80,81,82,83,84,85].

Through an extensive review of the extant literature, key elements of the studied phenomenon were delineated, along with potential interrelations among them. This process culminated in the development of a conceptual model designed to investigate the impact of culinary television programs on consumer behaviors, graphically depicted in Figure 1. 

This model posits that a consumer’s response may be triggered by a single exposure to a culinary program. Such a response typically involves visiting a featured establishment or one operated by a personality from the program. At these venues, consumer choices are driven by curiosity about flavors previously encountered only through the program. Concurrently, consumers immerse themselves in the ambiance of the venue, noting aspects such as décor, cleanliness, and service quality. This amalgamation of experiences evokes emotions, subsequently reflected in consumer opinions about the dish and establishment. These emotions are instrumental in shaping postconsumption feelings and overall consumer satisfaction. Depending on the nature and intensity of these emotions, consumers may choose to continue viewing culinary programs and may be inspired to attempt cooking these dishes themselves.

Comprehensive exploration of this phenomenon and the quantification of the impact exerted by culinary television programs on consumer behavior necessitate a series of methodological steps, which are detailed in the subsequent section of this article.

## 3. Materials and Methods

### 3.1. Research Methodology

**Figure 2:**
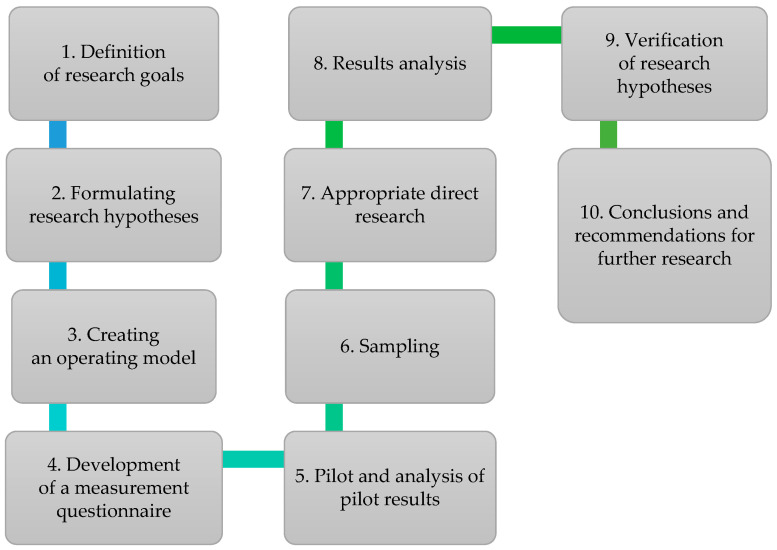
The research procedure. Source: own study based on [86].

The research procedure undertaken focused on a sequence of 10 stages, as delineated in Figure 2.

The development of the conceptual research model, its operationalization, and subsequent measurement were preceded by the articulation of research objectives and the formulation of hypotheses. Following the operationalization of the conceptual model, methods for selecting the research sample were established. This was succeeded by the execution of pilot studies and an analysis of the pilot data, to evaluate the relevance of incorporating specific variables into the model and to make necessary modifications. The finalized measurement model, encompassing both research constructs and indicator variables, facilitated the appropriate selection of the research sample, the execution of the primary study, and ultimately the collation and statistical analysis of the data. The culmination of this process was the verification of the proposed hypotheses and the formulation of recommendations for future research.

### 3.2. Objective of the Research and Hypotheses

The objective of the research was formulated as the identification of the impact of culinary television programs on consumer behavior in the gastronomic services market. The following research hypotheses were posited:

- H1: Culinary television programs determine consumer behaviors by influencing a higher frequency of using gastronomic services.
- H2: Consuming meals at restaurants recommended in culinary television programs affects consumers’ perception of their quality of life.
- H3: The impact of culinary television programs on the frequency of using gastronomic services is greater the higher are the consumers’ propensity for experimentation, motivations, curiosity about the world, and use of new technologies.

### 3.3. Operationalization of the Conceptual Research Model

Operationalization of the conceptual model represents a crucial stage in the research process, involving the translation of definitions and boundaries of theoretical constructs into the language of research operations. Operationalization occurs directly after conceptualization, which further specifies the terminology used in the studies [87]. Operationalization involves creating specific research procedures (operations) that will allow for empirical observations corresponding to conceptualized concepts in the real world [88].

Operationalizing the researched theoretical construct serves to build a measurement model, which plays a particularly important role in quantitative research [89]. Testing this model enables the verification of the assumed conceptual foundations, which aims to enrich existing theories. In quantitative research, the research model is the hypothetico-deductive model, which defines relationships between variables.

In the process of operationalization, the scope of variables in the model is defined, and their indicators are determined (translating concepts represented by variables into observable events that can be measured), designing questions (along with answer categories) used in measurement tools and determining methods and techniques of measurement and principles of variable analysis [90,91].

The phenomenon induced by the influence of the television program “Kitchen Revolutions” on consumer behavior in the gastronomic services market, consisting of the sensory experience of the quality of offerings from those gastronomic establishments that became “media known” through the program, has been termed “Gesslerism” (after the surname of the television program’s host). The authors are aware that they are introducing a new concept, but considering the scale of this phenomenon, they deemed it justified. The indicator of Gesslerism (G) is defined as the percentage of customers who visited the restaurant influenced by the “Kitchen Revolutions” program:

(1) $$G=\frac{n_{G}}{N}\cdot 100$$

where n_G_ is the number of people who came to the restaurant influenced by the “Kitchen Revolutions” program, and N is the number of people who visited the restaurant in the same period.

**Figure 3:**
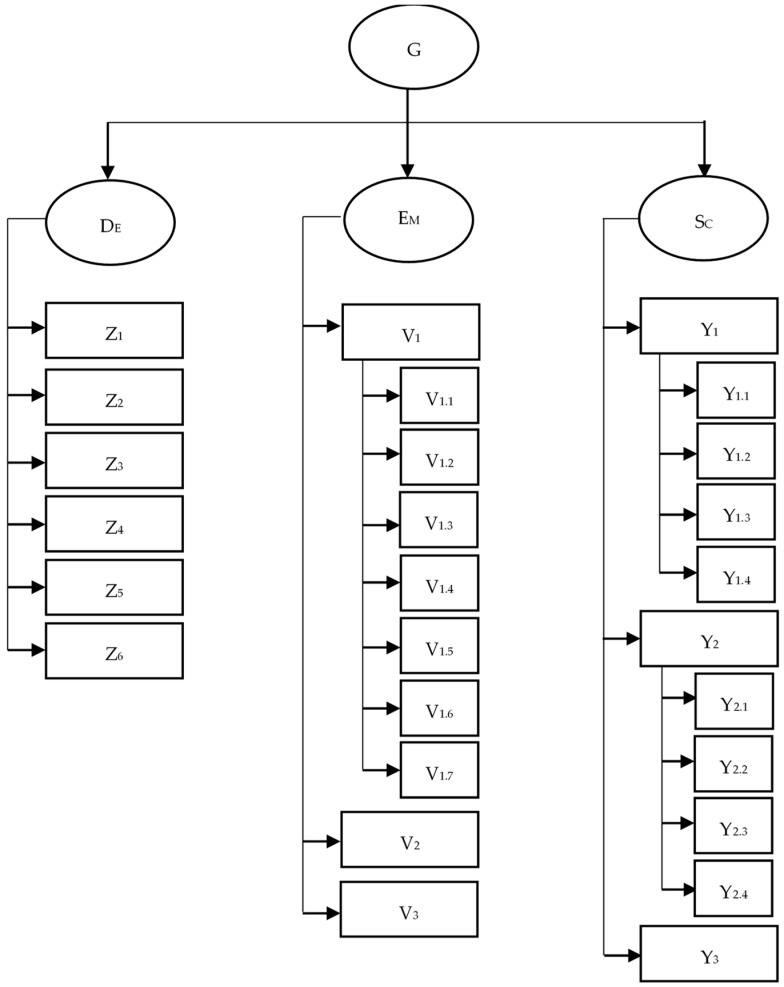
Operational model of the influence of culinary television programs on consumer behavior. Source: own study.

Gesslerism is characterized by the desire to experience the uniqueness of a restaurant D_E_, emotions E_M_, and sensations after consumption S_C_ (Figure 3). While the G index (1) is a directly measurable construct, the desire to experience the uniqueness of a restaurant D_E_, emotions E_M_, and sensations after consumption S_C_ are categories that are not directly observable (so-called latent [92]), which can only be measured indirectly through other variables (Z, V, Y).

The desire to experience the uniqueness of a restaurant is composed of variables such as quality of dishes (Z_1_), quality of service (Z_2_), cleanliness of the establishment (Z_3_), atmosphere of the establishment (Z_4_), decor of the establishment (Z_5_), and media uniqueness of the establishment (Z_6_). The emotion expression index (E_M_) is a set of variables: emotions after leaving the restaurant (V_1_), expressing opinions (V_2_), and the reach of these opinions (V_3_). In turn, the sensation after consumption index (S_C_) includes the following variables: feeling of satisfaction with the taste of the meal (Y_1_), feeling of satisfaction with the appearance of the meal (Y_2_), and perception of life quality resulting from the quality of consumed meals (Y_3_). A detailed breakdown of these variables is presented in Table 3.

### 3.4. Questionnaire Development

The next step in the operationalization process involved defining dependent and independent variables, along with selecting scales for their measurement. Dependent variables were identified for the four measurement indicators distinguished in Table 3, and questions for the measurement tool were designed. In these questions, various measurement scales were utilized: nominal, ordinal, and ratio scales.

An exploratory phase of research was conducted using the initial version of the questionnaire. The pilot study was conducted among 30 respondents using the face-to-face interview technique. These participants evaluated the instrument in terms of its substantive adequacy and pertinence, suggesting only marginal modifications to enhance its clarity and comprehension. After the pilot study, we checked the reliability of the research constructs. The Cronbach’s alpha for all latent variable groups was above the expected value of 0.7 and was, respectively, D_E_ = 0.823; V_1_ = 0.935; Y_1_ = 0.873, and Y_2_ = 0.892. The reliability indices obtained allowed the constructed research tool to be used for quantitative research.

### 3.5. Data Collections

The paper is based on the results of a questionnaire research study conducted with the computer-assisted web interviewing (CAWI) technique. Participants were assured of anonymity and confidentiality and were informed about the study’s scope and objectives in the introductory section of the survey. Participant identities and personal data were kept confidential throughout the research process. The nonrandom selection method was used for the selection of respondents for the study. The study population consisted of individuals who had watched the “Kitchen Revolutions” program on television at least once. The viewership figures for the last season of 27 aired from September to November 2023 indicate that the general population is 12.53% of Polish TV viewers in the 20–54 age group and 13.11% of viewers aged 16–49 (Table 4). 

The program evoked positive emotions and curiosity about the places the viewers had just seen on TV. The emotions were strong enough for them to decide to visit the restaurant they had seen. The selection criterion for the sample was visiting restaurants after watching the ‘Kitchen Revolutions’ program. The respondents were adults who consented to participate in the study. Participants in the survey visited a total of 92 restaurants that participated in the “Kitchen Revolutions” program in the years 2011–2022. The selected sample consisted of 846 respondents, among whom 742 who correctly completed questionnaires were analyzed. The survey was conducted between May 2021 and April 2022. The demographic characteristics of the survey participants are presented in Table 5.

## 4. Results

In the conducted research, results were obtained that allowed for the calculation of the Gesslerism index (G). By substituting into Formula (1) the number of people who visited restaurants influenced by the television program “Kitchen Revolutions” and dividing it by the number of people who visited these restaurants in total, a result of 74% was achieved. This indicates that nearly 3/4 of the respondents chose a given restaurant under the influence of this program. According to the authors, this result is impressive.

In line with the established assumptions, the phenomenon of Gesslerism is characterized by the desire to experience the uniqueness of a restaurant, emotions associated with consumption, and sensations after consumption.

Among the elements characterizing the uniqueness of the restaurant (D_E_ index), respondents rated the cleanliness, atmosphere, and decor of the restaurant the highest. The quality of dishes and that of service were also rated very highly. Nearly half of the respondents rated the media uniqueness of the restaurant at a medium level (Table 6).

The emotion expression index (E_M_) is composed of variables related to emotions experienced after leaving the restaurant. Participants who visited restaurants featured in “Kitchen Revolutions” predominantly felt a sense of satiation and believed that their time was very well spent, leading to significant satisfaction. After visiting the restaurant, respondents felt joy, were positively surprised by the visit, and mostly agreed that the taste of the ordered dishes was delightful. Furthermore, the participants considered that the visit to the restaurant after “Kitchen Revolutions” was worth its price (Table 7).

The emotion expression index also includes variables related to expressing opinions and the reach of these opinions. Almost all respondents (94.5%) expressed their opinion about their visit to the restaurant after “Kitchen Revolutions”. Nearly 70% of the participants talked about their restaurant visit with friends, almost 63% with family, and about 40% with work colleagues. A small percentage of the respondents declared posting their opinions on social media, Internet forums, or the restaurant’s website (Table 8).

**Figure 4:**
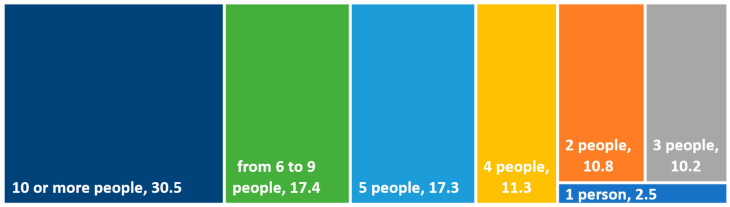
Number of people who were told by respondents about their emotions related to the restaurant visit (%). Source: own study.

The reach of the opinions expressed by respondents regarding their visit to a restaurant featured in “Kitchen Revolutions” is quite extensive. On average, respondents told about such a visit to seven people. Every tenth respondent shared their emotions and impressions from the restaurant visit with two, three, or four people. Nearly one-sixth of the respondents mentioned it to five people. Meanwhile, over 30% of the participants talked about such a visit to at least ten people (Figure 4).

The sensation after consumption index (S_C_) comprises variables related to the feeling of satisfaction with the taste of the ordered meal and the satisfaction with the appearance of the ordered meal. Both the taste and the appearance of the ordered dishes were rated very highly by the respondents. The taste was rated highest in the following order: main course, dessert, soup, and appetizer. Similarly, the appearance of the ordered dishes was rated as delightful in the order of main course, dessert, appetizer, and soup (Table 9).

**Figure 5:**
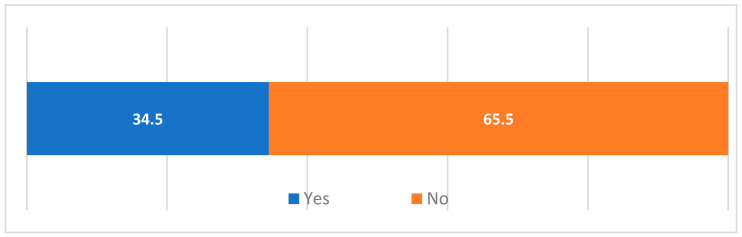
Declared influence of the “Kitchen Revolutions” program on increasing the frequency of dining out at restaurants (%). Source: own study.

The conceptual model of the impact of culinary television programs on consumer behavior in the gastronomic services market assumes, among other things, that such programs influence an increase in the frequency of dining out (hypothesis H1). These could be restaurants recommended by the program host, but they could also be more frequent visits to restaurants recommended by friends, family, and acquaintances. Only one in three respondents declared that the program “Kitchen Revolutions” influenced an increase in the frequency of dining out (Figure 5). However, respondents more frequently visit restaurants recommended by friends and acquaintances (63% of indications) than those recommended by Magda Gessler (46% of indications). Furthermore, every third respondent who dines out more frequently does not follow any recommendations at all.

Considering that in the set of determinants influencing consumer choices in the gastronomic services market there are many factors (including location, prestige, type of cuisine, prices, reviews, information) [95,96,97,98,99], and their significance ranges from a few to several tens of percent, the importance of a factor such as Gesslerism can be considered significant. 

**Figure 6:**
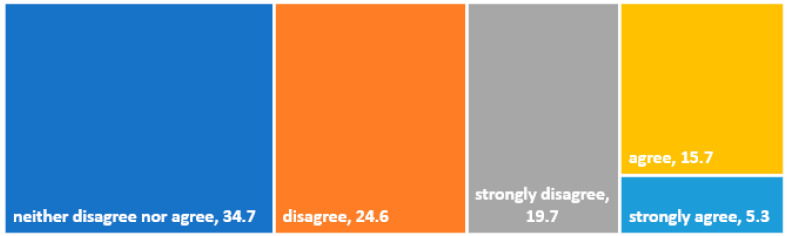
Opinions on the statement: eating at restaurants recommended by Magda Gessler influences the quality of my life (%). Source: own study.

The authors of the research model assumed that dining at a restaurant featured in a culinary TV show would be a significant emotional experience. Experiencing the highest level of gastronomy, guaranteed by Magda Gessler, could influence the feeling of a “higher quality of life” (hypothesis H2). However, the results obtained did not confirm this hypothesis. While one in five respondents agreed with the statement “Eating at restaurants recommended by Magda Gessler influences the quality of my life”, the majority disagreed or had no opinion on this matter at all (Figure 6). The results indicated that for Polish consumers, the feeling of satiation, the cleanliness of the establishment, and its decor are more important than the quality of the meal and the emotions resulting from the media uniqueness of the establishment.

The independent variables in the model of the impact of culinary television programs on consumer behavior in the gastronomic services market, apart from demographic characteristics, include personality traits. The set of personality traits examined encompassed propensity for experimentation, interest in technology, level of motivation (engagement and goal achievement), and curiosity about the world. The study participants are individuals curious about the world, many of them set goals and achieve them, a large portion like to experiment, nearly as many like to engage, and they belong to the group of new technology enthusiasts (Table 10).

The authors hypothesized that the impact of culinary television programs on the frequency of using gastronomic services is greater the higher the consumers’ propensity for experimentation is, the stronger motivations are, and the greater the curiosity about the world is, as well as greater use of technology is (H3). The obtained correlation statistics in this regard only indicate a statistically significant relationship between the variable “frequency of using gastronomic services” and the variable “propensity for experimentation” (Table 11). The greater is the propensity for experimentation among the study participants, the more willingly and frequently they dine out. The other personality traits do not significantly influence the frequency of dining out among the respondents.

The analyzed variables in the study are interconnected in several ways, revealing the complex dynamics of consumer behavior in the gastronomy services market and the influence of culinary TV programs. The emotional responses, such as delight or disappointment in the taste and appearance of dishes, influence the overall dining experience, contributing to shaping the frequency of dining out. The sensation after consumption index, which incorporates satisfaction with taste and appearance, reflects the overall positive or negative sentiments after dining and is influenced by dish ratings for taste and appearance. The study explores the impact of culinary TV programs, specifically “Kitchen Revolutions”, on the frequency of dining out, indicating a moderate influence on actual dining habits. Personal recommendations from friends and family have a stronger influence on dining frequency compared with recommendations from culinary TV programs, with a significant portion of individuals relying on personal networks for making dining choices.

The study also investigates whether dining at restaurants recommended by culinary TV programs affects the perceived quality of life, showing limited agreement among respondents and suggesting that factors like satiety, cleanliness, and ambiance are more influential. The study also examines personality traits, including the propensity for experimentation and interest in technology, and their correlation with dining choices. Individuals with a higher propensity for experimentation are more likely to dine out frequently, indicating a connection between personality traits and dining behavior. These connections emphasize the multifaceted nature of the variables, where emotional experiences, external influences like TV programs and personal recommendations, and individual traits collectively contribute to shaping consumer decisions in the gastronomy services market.

## 5. Discussion

The study set out to investigate the impact of culinary TV programs, such as “Kitchen Revolutions”, on the dining-out frequency of consumers. According to the uses and gratifications theory [100,101], viewers engage with media for various reasons, including information, entertainment, personal identity, and integration. In the context of culinary TV programs, viewers might seek both entertainment and information about dining options. However, the study’s data suggest that only 32.3% of the respondents reported an increase in their dining-out frequency after watching the program.

Ngqangashe et al. [102] used the gratification theory for the analysis of food media consumption among adolescents. Comparing with the research presented in this paper, adolescents consume food media both incidentally and selectively. TV cooking shows are more often incidentally consumed, while online food media is more selectively sought. Different motives drive the consumption of food media. TV cooking shows are primarily consumed for companionship and entertainment, while online food media serves a more diverse range of motives, with a focus on information, inspiration, and social interaction. This study also sheds light on different aspects of food media consumption and provides insights into the motivations and effects of engaging with food-related content. Some of those effects, such as looking for entertainment, are similar.

The data illustrate the variance in responses. It is noteworthy that the delightful taste of the appetizer received a high rating (43.9%), indicating that viewers may derive enjoyment from watching the show and experiencing the featured cuisine. However, the translation of this enjoyment into a significant change in dining-out behavior was limited. This result aligns with the uses and gratifications theory, as the program appears to gratify the viewers’ need for entertainment and culinary information but does not lead to a substantial behavioral change. The importance of delightful taste in a restaurant was also found out as an important factor by Barnes et al. [103], which was also reported by Kim et al. [104].

The research presents the ratings of emotions after leaving the restaurant. These emotions are related to the taste and look of different dishes. The results show that there is a significant variation in the emotional responses of diners, both positive and negative. This indicates that the quality of the dining experience can vary widely and may depend on individual preferences. For instance, when it comes to the taste of the dishes, some diners reported disappointment, while others found the tastes delightful. This suggests that different consumers have different expectations and preferences when dining out. The results underline the subjectivity of taste and the challenge for restaurants in meeting diverse customer expectations. Many other researchers also pointed out the role of emotion in restaurant service [105,106,107] and differentiation in their emotional response [108,109].

Similarly, the emotions related to the look of dishes vary, indicating that the visual presentation of food is also an important aspect of the dining experience. While some customers may be disappointed with the look of their dishes, others may find them delightful. Findings highlight the complex and nuanced nature of customer emotions and the need for restaurants to cater to a wide range of tastes and preferences.

The research hypothesizes that such programs would increase the frequency of dining out. However, the results show that only a third of the respondents claimed that the program influenced them to dine out more frequently. Interestingly, a higher percentage of the respondents reported being influenced by recommendations from friends and family compared with those influenced by Magda Gessler’s recommendations. A significant portion of those who dine out more frequently do not base their choices on any recommendations at all. These findings suggest that while culinary TV shows may have an impact, word-of-mouth recommendations from personal networks play a more significant role in shaping consumer behavior in the restaurant industry. The problem was also analyzed by Curnutt [110] and Hollow and Jones [111]. In those papers, the rising role of cooking programs as a factor shaping consumers behavior could be observed.

Additionally, the research investigated whether dining at restaurants featured on TV shows, especially those recommended by Magda Gessler, had a positive impact on the quality of life. The results indicate that this was not the case for most respondents. Only a minority agreed that dining at such restaurants improved their quality of life. For the majority of consumers, factors like satiety, cleanliness, and restaurant ambiance seemed to be more important than media exposure and celebrity endorsements.

The study also explored the relationship between personality traits and dining habits. The existence of this relationship has been highlighted by researchers, such as Golestanbagh et al. [112] and Goldberg [113]. Personality traits taken into account encompass a disposition for experimentation, enthusiasm for technology, motivation levels, and curiosity. The results showed a significant positive correlation between the propensity for experimentation and the frequency of dining out. This suggests that individuals with a higher inclination to try new things are more likely to dine out frequently. Additionally, a positive correlation between eating out and interest in technology was observed. One possible explanation for the observed positive correlation between eating out and an interest in technology could be the increasing integration of technology in the restaurant industry. Technology-driven advancements, such as online reservations, mobile ordering, digital menus, or interactive dining experiences, may attract individuals with a keen interest in technology. People who enjoy exploring and adopting new technological tools and innovations may find the tech-enhanced aspects of dining out appealing, contributing to the observed correlation.

In contrast, other personality traits such as motivation and curiosity did not have a significant influence on dining frequency.

The theory of parasocial interaction [114,115] posits that viewers develop emotional connections with media personalities or characters. In the case of culinary TV programs, such as “Kitchen Revolutions”, viewers may form parasocial relationships with the hosts, like Magda Gessler, which can affect their perceived quality of life. However, the study results do not strongly support this hypothesis. Only 20% of the respondents agreed with the statement that eating at restaurants recommended by Magda Gessler influenced the quality of their lives.

The study revealed that emotional responses to culinary TV programs can be substantial. For instance, in our survey, approximately 43.9% of the respondents reported delight in the taste of the appetizer after watching the program, while 33.4% found the dessert’s taste delightful. On the contrary, 20.6% of the respondents were disappointed with the appetizer’s taste, and 28.0% were disappointed with the soup’s taste. The research underscores that while emotional engagement with the TV program may occur, it does not necessarily translate into a significant influence on the overall quality of life. This is consistent with the parasocial interaction theory, as the emotional connection established with Magda Gessler or the show does not profoundly affect the respondents’ daily lives.

The data revealed a significant correlation between the respondents’ inclination to experiment and their dining choices. This finding can be linked to the big five personality traits theory, which includes the trait of openness to experience. Individuals scoring high on openness to experience are more likely to be open to trying new things, which, in this context, is reflected in their dining choices.

Our research demonstrates the significant correlation between the inclination to experiment and dining frequency. This supports the notion that personality traits can influence the impact of culinary TV programs, with individuals open to experimentation being more influenced by the program.

The study’s findings provide valuable insights within the context of media theories and personality traits. They suggest that the influence of culinary TV programs on dining behavior and perceived quality of life is nuanced and multifaceted. While viewers may form emotional connections with the program or its hosts, the translation of these emotions into significant behavioral changes is not guaranteed. Additionally, personality traits, such as the inclination to experiment, play a role in how viewers respond to these programs. These results can be seen as a complex interplay between media influence, emotions, and individual characteristics, aligning with various communication and psychological theories [116,117,118].

There could also be various contextual factors influencing changes in consumer behavior beyond the scope of the culinary TV programs discussed in the paper. Economic conditions, such as recessions or economic growth, can significantly impact consumers’ disposable income and, consequently, their dining-out habits [104]. During economic downturns, consumers might opt for more cost-effective dining options or reduce their overall frequency of dining out [104].

Shifts in cultural preferences and social trends can influence consumers’ choices. For example, increasing health consciousness might lead to a rise in demand for healthier dining options, affecting where and how often individuals choose to eat out [105]. Ongoing technological advancements in the restaurant industry, such as the widespread adoption of online ordering platforms, contactless payments, or innovative dining experiences, can influence consumer preferences and dining habits [106].

Major global events, such as pandemics or geopolitical crises, can have a profound impact on consumer behavior. The COVID-19 pandemic, for instance, led to widespread changes in dining habits, with an increased reliance on takeout and food delivery services [119]. Moreover, there are difference types of consumers in the market. Some make decisions influenced by emotions, others by budgets, and still others are conscious of sustainable consumption [120]. There are also stereotypical consumers on the market who have not changed their preferences for years. Gastronomy developed under the influence of television programs is a new form of influencing the tastes and preferences of consumers. Effective marketing and advertising campaigns can influence consumer perceptions and choices [107]. Promotions, loyalty programs, and advertising strategies by restaurants may impact consumer decisions on where and how often to dine out [108]. Changes in food safety regulations, health standards, or restaurant licensing requirements can affect the choices consumers make. Compliance with new regulations may influence the perceived trustworthiness and desirability of certain dining establishments [109].

## 6. Conclusions

This study examined the influence of culinary TV programs, with a specific focus on “Kitchen Revolutions”, on consumer behavior in the gastronomy services market. It delved into the realm of emotions, personality traits, and dining choices to unravel the complexities of this relationship.

The study also examined personality traits and found that the inclination to experiment, a trait indicating a willingness to try new experiences, had a significant influence on dining choices. Respondents with a higher inclination to experiment were more likely to dine out frequently.

The hypothesis H1 that culinary TV programs would increase the frequency of dining out, in restaurants recommended by either the TV hosts or by friends and family, was partially supported. 

The hypothesis H2 positing that dining at establishments featured in these TV shows, often associated with celebrity chefs like Magda Gessler, would influence consumers’ perceptions of life quality was not strongly supported. Approximately 20% of the participants agreed that eating at these restaurants influenced the quality of their lives.

The hypothesis H3 suggesting that the influence of culinary TV programs would be greater among consumers with higher levels of certain personality traits was only partially supported. The inclination to experiment was found to be significantly correlated with dining choices, but other personality traits did not have a substantial impact.

Results offer several important implications for the restaurant industry. First, understanding the emotional impact of dining experiences is crucial. Restaurants should focus on consistently delivering high-quality food and an appealing presentation, as these factors directly influence customer satisfaction.

Regarding the influence of culinary TV shows, while they can attract customers, the power of personal recommendations cannot be underestimated. Restaurants should encourage positive word-of-mouth marketing and reviews from satisfied customers.

The study’s findings on personality traits suggest that targeting experimental consumers could be a potential marketing strategy for restaurants. Restaurants may consider offering unique and innovative dining experiences to attract this segment of customers.

The main scientific value of this paper lies in its contribution to understanding the complex interplay of factors that influence consumer behavior in the gastronomy services market, particularly the influence of culinary TV programs and the role of emotional responses and personality traits. It could be stated that the scientific value of this paper is its holistic exploration of factors influencing consumer behavior in the gastronomy services market, its empirical nature, and the insights it offers to enhance our understanding of how customers make dining choices. These findings can inform not only academic discussions but also practical strategies for restaurants and marketers in the culinary industry.

## 7. Limitations and Future Research

A significant limitation of the field research was the timing of its execution, which coincided with the COVID-19 pandemic period. The authors initially planned to conduct the research using face-to-face interview techniques. However, the restrictions on the operation of restaurants due to the lockdown necessitated the implementation of the studies using the CAWI technique. The data collected for the study rely on self-reported responses from participants. Self-reporting can introduce response bias, as participants may not always provide completely accurate or objective information about their dining experiences. The paper primarily centers around the influence of the “Kitchen Revolutions” program. While this program is popular, studying the effects of a broader range of culinary TV shows would provide a more comprehensive understanding of the media’s impact. 

Several other limitations should be acknowledged based on our restaurant-focused research. It should be mentioned that a noteworthy observation is the over-representation of female respondents in our study. This gender imbalance raises questions about the generalizability of our findings and whether it may have influenced the outcomes. For example, if there are gender-specific trends in dining habits or preferences, the prevalence of female respondents could potentially skew the results. Additionally, factors like a higher proportion of females watching TV shows might introduce a bias, potentially impacting the patterns depicted in Figure 5.

Furthermore, the decision-making process regarding dining out was not extensively explored in our research. Understanding who makes the decisions to dine out and the factors influencing those decisions could provide valuable insights. Exploring this aspect might uncover nuances in the relationship between various variables and restaurant-related behaviors.

Demographic distributions, beyond gender, were not deeply delved into, and these could introduce confounding variables. Differences in age, income, or cultural background might play a role in shaping attitudes and behaviors related to restaurant choices.

Future research could employ longitudinal designs to track changes in consumer behavior over an extended period. This would help in understanding the long-term impact of culinary TV programs on dining choices and whether any influence diminishes or intensifies over time. Expanding the scope to include a broader range of culinary TV programs would be valuable. A comparative analysis could explore how different program styles, hosts, and contents affect consumer behavior. Examining international shows and their impact on local dining choices could be of particular interest.

## Figures and Tables

**Table 2:** Factors influencing choice of gastronomic local by consumers.

Factors	Description
Celebrity chef endorsement	Viewers are often drawn to restaurants featured or endorsed by famous chefs seen on TV.
Food presentation on TV	Visually appealing dishes showcased on television can pique viewers’ interest in trying them in person.
Unique and exotic ingredients	TV programs often introduce novel or exotic ingredients, sparking curiosity among viewers to taste them.
Host recommendations	If the TV host recommends a specific restaurant or dish, viewers tend to trust their judgment and visit.
Location and ambiance	The location and ambiance portrayed on TV can attract viewers who seek a similar dining atmosphere.
Positive reviews and ratings	Favorable reviews and ratings mentioned on TV programs can influence people’s dining choices.
Cultural exploration	Culinary shows that explore diverse cuisines and cultures inspire viewers to try new food experiences.
Social media influence	Social media buzz and trends related to culinary TV programs can lead people to featured restaurants.
Dietary compatibility	Some viewers choose restaurants aligned with their dietary restrictions or preferences as seen on TV.
Emotional connection	TV programs that evoke emotions or nostalgia can motivate viewers to visit places associated with those feelings.
Accessibility and convenience	Proximity and ease of access to the featured restaurant play a role in the decision-making process.
Budget and affordability	The cost of dining at a featured restaurant may impact the choice, depending on one’s budget.
Word of mouth	Recommendations from friends and family who watched the same TV show can strongly influence choices.
Special offers and promotions	Discounts or promotions offered by restaurants featured on TV can attract viewers.

Source: own study based on [1,2,3,33,34,35,40,41,43,46].

**Table 3:** Dependent and independent variables of the model of the impact of culinary television programs on consumer behavior.

Specification	Element of the Operationalization Process
Dependent variables	1. Variables for the G indicator: Did you visit the restaurant influenced by the “Kitchen Revolutions” program? (response: yes/no) 2. Variables for the D_E_ indicator: (Z_1_) How do you rate the quality of the dish ordered (from Magda Gessler’s menu)? (response on a scale of 1 to 5, where 1 is very low, 5 is very high) (Z_2_) How do you rate the quality of service? (response on a scale of 1 to 5, where 1 is very low, 5 is very high) (Z_3_) How do you rate the cleanliness of the establishment? (response on a scale of 1 to 5, where 1 is very dirty, 5 is very clean) (Z_4_) How do you rate the atmosphere of the establishment? (response on a scale of 1 to 5, where 1 is very unpleasant, 5 is very pleasant) (Z_5_) How do you rate the decor of the establishment? (response on a scale of 1 to 5, where 1 is very ugly, 5 is very nice) (Z_6_) How do you rate the media uniqueness of the establishment? (response on a scale of 1 to 5, where 1 is very low, 5 is very high) 3. Variables for the E_M_ index: (V_1_) What emotions did you feel after leaving the restaurant? (answers on a scale of 1 to 5) (V_1.1_) Negative surprise—Positive surprise; (V_1.2_) Disappointment—Satisfaction; (V_1.3_) Sadness—Joy; (V_1.4_) Regret spending money—Visit worth its price; (V_1.5_) Visit was a waste of time—Time well spent; (V_1.6_) Felt underfed—Felt very full; (V_1.7_) Disappointed by the taste of ordered dishes—Delighted by the taste of ordered dishes (V_2_) Whom did you tell about your emotions related to the restaurant visit? (answer: family, friends, work colleagues, wrote a review on social media, wrote a review on an Internet forum, wrote a review on the restaurant’s website, other ways, which?) (V_3_) How many people did you tell about your emotions related to the restaurant visit? 4. Variables for the S_C_ index: (Y_1_) How do you rate the taste of the ordered dishes? (Y_1.1_) Disappointed by the taste of the ordered starter—Delighted by the taste of the ordered starter; (Y_1.2_) Disappointed by the taste of the ordered soup—Delighted by the taste of the ordered soup; (Y_1.3_) Disappointed by the taste of the ordered main dish—Delighted by the taste of the ordered main dish; (Y_1.4_) Disappointed by the taste of the ordered dessert—Delighted by the taste of the ordered dessert (Y_2_) How do you rate the appearance of the ordered dishes? (Y_2.1_) Disappointed by the appearance of the ordered starter—Delighted by the appearance of the ordered starter; (Y_2.2_) Disappointed by the appearance of the ordered soup—Delighted by the appearance of the ordered soup; (Y_2.3_) Disappointed by the appearance of the ordered main dish—Delighted by the appearance of the ordered main dish; (Y_2.4_) Disappointed by the appearance of the ordered dessert—Delighted by the appearance of the ordered dessert (Y_3_) Do you agree with the following statement (on a scale of Strongly disagree to Strongly agree): Eating meals at restaurants recommended by Magda Gessler affects the quality of my life.
Independent variables	1. Personality profile Please rate on a scale of 1 to 5 your approach to life: I like to experiment, I am a fan of technology, I like to engage, I set goals for myself and achieve them, I am curious about the world. 2. Demographic profile Gender (female, male), Age, Education (primary, vocational, secondary, higher)

Source: own study.

**Table 4:** Viewership indicators of the “Kitchen Revolutions” program.

Indicators		Season 25 (from September 2022 to December 2022)	Season 27 (from September 2023 to October 2023)
Viewers aged 4+	AMR *	1,304,732	1,082,940
	SHR % **	12.01%	10.09%
	RCH (1 min) ***	2,459,282	2,010,720
Viewers aged 16–49	AMR	620,573	496,222
	SHR %	15.89%	13.11%
	RCH (1 min)	1,119,977	869,494
Viewers aged 20–54	AMR	243,017	574,130
	SHR %	17.88%	12.53%
	RCH (1 min)	405,484	1,027,244

Source: own study based on [93,94]. * AMR (average minute rating) is the average minute viewership—an indicator describing the average size of the audience watching a specific program or television program over any specified period of time. ** SHR% (audience share) is the audience share of a given channel in the TV market, i.e., the percentage of viewers who watched a given program in relation to all viewers who watched TV in a given period. *** RCH (reach) is the number of viewers who watched at least 1 minute of a given program or had contact with a TV station during the entire period of time studied.

**Table 5:** Sample characteristics.

Characteristics	Item	%
Gender	Female	61.6
	Male	38.4
Age (years)	18–24	29.0
	25–29	28.6
	30–39	19,4
	40 and more	23.0
Education	Primary	1.4
	Vocational	7.5
	Secondary	35.6
	Higher	55.5

Source: own study.

**Table 6:** Opinions on experience of the uniqueness of the restaurant (%).

Items	1	2	3	4	5
Quality of the food	1.4	5.2	24.0	51.0	18.4
Quality of the service	1.9	6.5	22.7	51.2	17.7
Cleanliness	1.0	1.6	10.4	62.8	24.3
Ambience	0.3	3.3	12.1	60.2	24.2
Design	0.7	5.4	17.4	57.3	19.3
Uniqueness	3.0	11.8	46.1	29.2	9.9

Source: own study. A 5-point scale in which 1 is the lowest rating and 5 is the highest rating.

**Table 7:** Rating of emotions after leaving the restaurant (%).

	1	2	3	4	5	
Negative surprise	2.8	6.5	23.6	40.6	26.5	Positive surprise
Disappointment	4.7	6.9	13.6	39.1	35.7	Satisfaction
Sadness	3.4	3.0	19.0	41.4	33.2	Joy
Regret for money spent	6.4	6.6	20.1	34.2	32.7	A visit worthwhile
The visit was a waste of time	4.9	3.7	14.7	33.9	42.8	Time very well spent
Feeling unfulfilled	3.0	4.5	14.7	34.0	43.8	Feeling very full
Disappointed with the taste of the food	5.4	5.3	15.3	47.8	26.2	Delightful taste of the food

Source: own study. A 5-point scale in which 1 is the lowest rating and 5 is the highest rating.

**Table 8:** People who were told by respondents about their emotions related to the restaurant visit.

Items	%
Friends	69.9
Family	62.9
Colleagues at work	38.3
Social media opinion	7.1
Internet forum	4.0
Restaurant website review	3.4

Source: own study.

**Table 9:** Rating of taste and look of the ordered dishes (%).

	1	2	3	4	5	
The appetizer’s taste disappointed me	4.5	5.9	20.6	43.9	25.1	The appetizer’s taste was delightful
The soup’s taste disappointed me	3.7	6.0	18.8	43.5	28.0	The soup’s taste was delightful
The main course’s taste disappointed me	4.0	4.7	14.9	44.0	32.3	The main course’s taste was delightful
The dessert’s taste disappointed me	2.7	4.5	17.6	41.8	33.4	The dessert’s taste was delightful
The appetizer’s look disappointed me	2.3	4.9	17.6	48.3	26.8	The appetizer’s look was delightful
The soup’s look disappointed me	2.8	5.1	21.2	45.6	25.3	The soup’s look was delightful
The main course’s look disappointed me	2.8	2.9	16.1	48.0	30.2	The main course’s look was delightful
The dessert’s look disappointed me	2.5	3.1	18.3	41.9	34.1	The dessert’s look was delightful

Source: own study.

**Table 10:** Personality traits of respondents (columns 1–5 in %).

Items	1	2	3	4	5	Mean	Median	Items
I do not like to experiment	2.8	4.3	19.3	31.7	41.9	4.05	4.0	I like to experiment
I am not a fan of technology	4.3	6.2	23.5	35.2	30.7	3.81	4.0	I am a fan of technology
I do not like to engage	4.2	6.5	21.0	33.3	35.0	3.88	4.0	I like to engage
I do not set goals for myself	2.6	3.7	18.4	36.2	39.2	4.06	4.0	I set goals for myself and achieve them
I am not curious about the world	2.0	1.4	9.1	25.6	61.9	4.44	5.0	I am curious about the world

Source: own study.

**Table 11:** Statistics of the relationship between the frequency of using gastronomic services and the personality traits of the respondents.

Traits	Pearson’s Chi-Squared Test	df	Asymptotic Significance (Two-Sided)
Propensity for experimentation	19.625	4	<0.001
Interest in technology	13.161	4	0.011
Engagement	5.534	4	0.237
Achieving goals	9.749	4	0.045
Curiosity about the world	8.410	4	0.078

Source: own study.

## Data Availability

The data presented in this study are available on request.
